# Comparison of MRI T1, T2, and T2* mapping with histology for assessment of intervertebral disc degeneration in an ovine model

**DOI:** 10.1038/s41598-022-09348-w

**Published:** 2022-03-30

**Authors:** Nora Bouhsina, Cyrille Decante, Jean-Baptiste Hardel, Dominique Rouleau, Jérôme Abadie, Antoine Hamel, Catherine Le Visage, Julie Lesoeur, Jérôme Guicheux, Johann Clouet, Marion Fusellier

**Affiliations:** 1 INSERM, UMRS 1229, Regenerative Medicine and Skeleton (RMeS), Université de Nantes, ONIRIS, 44042 Nantes, France; 2 Université de Nantes, UFR Odontologie, 44042 Nantes, France; 3 Department of Diagnostic Imaging, CRIP, ONIRIS, College of Veterinary Medicine, Food Science and Engineering, 44307 Nantes, France; 4 Service de Chirurgie Infantile, PHU5, CHU Nantes, 44093 Nantes, France; 5 Laboniris, ONIRIS, College of Veterinary Medicine, Food Science and Engineering, 44307 Nantes, France; 6 CRCINA, INSERM, Université d’Angers, Université de Nantes, Nantes, France; 7 PHU4 OTONN, CHU Nantes, 44093 Nantes, France; 8 UFR des Sciences Biologiques et Pharmaceutiques, Université de Nantes, 44042 Nantes, France; 9 Pharmacie centrale, PHU11, CHU Nantes, 44042 Nantes, France

**Keywords:** Medical research, Pathogenesis, Rheumatology

## Abstract

An easy, reliable, and time-efficient standardized approach for assessing lumbar intervertebral disc (IVD) degeneration with relaxation times measurements in pre-clinical and clinical studies is lacking. This prospective study aims to determine the most appropriate method for lumbar IVD degeneration (IDD) assessment in sheep by comparing three quantitative MRI sequences (variable-flip-angle T1 mapping, and multi-echo T2 and T2* mapping), correlating them with Pfirrmann grading and histology. Strong intra- and interrater agreements were found for *Nucleus pulposus* (NP) regions-of-interest (ROI). T1, T2, and T2* mapping correlated with Pfirrmann grading and histological scoring (p < 0.05) except for the most ventral rectangular ROI on T2 maps. Correlations were excellent for all of the T1 ROIs and the T2* NP ROIs. Highly significant differences in T1 values were found between all Pfirrmann grades except between grades I/II and between grades III/IV. Significant differences were identified in the T2 and the T2* values between all grades except between grades I/III. T1, T2, and T2* relaxation times measurements of the NP are an accurate and time-efficient tool to assess lumbar IDD in sheep. Variable-flip-angle T1 mapping may be further considered as a valuable method to investigate IDD and to assess the efficacy of regenerative treatments in longitudinal studies.

## Introduction

Low back pain (LBP) is a major health concern that affects a great part of the population^1^ and has a real socio-economic impact^2,3^. Numerous mechanisms have been studied, and lumbar intervertebral disc degeneration (IDD) has been identified as one of the main causes of non-specific LBP^4,5^. Biochemical changes occur early in the degenerated intervertebral disc (IVD) with proteoglycan loss, dehydration, and collagen fibers degradation especially in the *Nucleus pulposus* (NP)^6^. These modifications lead to morphologic changes as well as biomechanical and functional failures, some factors which may cause discogenic back pain.

Image-based assessment of the IVD is essential to determine the severity of IDD in vivo and to select the most suitable patient management. Numerous grading systems have been developed to score the degeneration of the IVD or the facet joints^7^. Radiologic features of the IVD have been historically well-studied as being the first imaging modality widely accessible. But by its entire soft tissue opacity, the different parts of the IVD are not distinguishable, in particular in the early stages of the degeneration process. The radiographic signs of IDD are mainly the disc height loss with a narrowing of the IVD space. An increased radio-opacity may sometimes be observed on the projection area of the NP, representing intradiscal calcification. Sclerosis of the endplates and osteophytes can also be associated with the degenerative process^8^.

Over the past fifteen years, magnetic resonance imaging (MRI) has been recognized as the most useful imaging modality to investigate and characterize IDD. This non-invasive cross-sectional imaging technic has an important soft tissue contrast which allows a good distinction between the NP and the *Annulus fibrosus* (AF) in a healthy tissue. On the contrary, even if the IVD can be assessed in the three dimensions, computed tomography meets the same contrast restrictions as radiographs and is however less relevant than MRI to investigate the changes which occur in the IVD. MRI allows mainly qualitative evaluations of the IVD. Among these evaluations, the Pfirrmann grading system for T2-weighted MRI conventional sequences evaluates the signal intensity and homogeneity of the NP as well as its transition zone with the AF and the variation of the IVD height^9^. Although Pfirrmann grades are commonly used to assess IDD, there remains a degree of subjectivity and observer bias. This scoring system also fails to detect subtle degenerative changes that arise in the IVD, which can, however, be instrumental to assess the efficacy of a therapeutic strategy in longitudinal studies^7,10^.

Quantitative MRI, and in particular relaxation time measurements also called mapping, has been recently developed to characterize more precisely the variations in the tissue composition of the IVD, the cartilage, or the brain^11–13^. IDD implies alterations of the biochemistry and the molecular environment and interactions which can be detected on MRI mapping sequences. These sequences aim to provide absolute values that may be useful for detecting subtle variations in the considered tissue or for longitudinal studies.

T2 mapping reflects the network of proteoglycan fibers, the water content, as well as molecular interactions, which tend to decrease with IDD^11,14^. T2* mapping allows assessment of the ultrastructural tissue composition of the IVD by providing information regarding the spatial architecture of the macromolecules and the mobility of water molecules^11,12^, and it correlates with functional lumbar mechanics^10^. T1 mapping has been widely used in cartilage degeneration^15,16^, brain sclerosis^17^, or cardiovascular studies^18^ and has been shown to be correlated to the water content^16^. Nevertheless, its usefulness remains to be evaluated in IDD and particularly on the NP.

Few studies have correlated the T1, T2, and T2* relaxation time values and the histological data^19–22^. NP histological evaluation is the gold standard to assess the tissue changes in the lumbar IVD thus assessing the correlation between the two parameters appears to be particularly relevant. All of the studies which have correlated MRI relaxation sequences with histology used animal models. Surprisingly, whereas sheep is one of the most frequently used large animal models for assessing IDD^23–26^, only one study has been carried with this species to date^19^.

Development of MR mapping sequences aims to evaluate their pertinence in order to apply them in routine clinical evaluation. Yet, some MRI sequences investigated in fundamental research projects are not available on standard clinical MR scanners or not feasible on patients because of their long time of acquisition. In routine, clinicians need MR sequences and measurement methods that are readily easy to use, reliable, repeatable, specific, and not time-consuming to assess IDD. The time dedicated to one patient depends mostly on (1) the time of acquisition of the different sequences which need to be as shorter as possible to get an image quality acceptable for the analysis and (2) the method initially chosen for image analysis. Various methods of region-of-interest (ROI) drawing have been described to define the NP, the AF, or the junctions and to measure the relaxation times of the studied parts of the IVD but no consensus has been found. Only one study has compared three different approaches to define the different parts of the human IVD on T2 and T1rho mapping sequences and no real differences have been found between the manual method and the 5 or 7-squares delimitations of the IVD^27^.

Thus, the objectives of this study were (1) to compare MRI T1, T2, and T2* mapping using two region-of-interest (ROI) drawing methods on lumbar ovine IVD to determine the most suitable approach to assess the changes occurring during the degeneration process and that could be used in preclinical and clinical investigations, and (2) to correlate the MR relaxation times with the histological scoring to establish whether MR mapping sequences may be useful in longitudinal studies for assessing therapeutic strategies in IDD.

## Methods

### Cohorts

Fifty lumbar IVDs of 10 sexually mature healthy female sheep between 24 and 108 months of age (mean age: 50.4 months) were prospectively included. None of the animals exhibited musculoskeletal or neurological conditions or signs of low back pain. The cohort came from an accredited farm (GAEC HEAS farm, Ligné, France). This study was approved by the French Ministry of Agriculture (Ethics Approval number: CEEA-PDL APAFIS 6170). All of the animal experiments were carried out in accordance with EU Directive 2010/63/EU and ARRIVE guidelines.

### Image acquisition

The animals were anesthetized prior to the imaging procedures. The anesthesia was maintained by inhalation of isoflurane (1–3%) after intravenous injection of diazepam (0.2–0.4 mg/kg), ketamine (2–5 mg/kg), and propofol (1–3 mg/kg).

Frontal and mid-sagittal radiographs of the lumbar spine were performed using a collimator-to-film distance of 100 cm, exposure of 20–30 mAs, and penetration power of 70–85 kV (Convix 80 generator, Universix 120 table, USA).

MR images were obtained on the lumbar spine of each sheep with a 1.5 T MR scanner (Magnetom-Essenza, Siemens Medical Solutions, Germany) and a standard spine coil. Sagittal T1-weighted fast spin-echo images (repetition time: 322 ms; echo time: 12 ms; flip angle: 120°; thickness: 3 mm; field of view: 512 × 512; bandwidth: 200; pixel size: 0.83 × 0.83 mm) and sagittal T2-weighted fast spin-echo images (repetition time: 3000 ms; echo time: 86 ms ; flip angle: 120°; thickness: 3 mm; field of view: 512 × 512; bandwidth: 200; pixel size: 0.83 × 0.83 mm) were acquired. These sequences were followed by the acquisition of sagittal (1) variable flip-angle T1 mapping sequence (repetition time: 15 ms; echo time: 1.7 ms; flip angle: 5°/26°; thickness: 3 mm; field of view: 256 × 264; bandwidth: 280; pixel size: 1 × 1 mm), (2) multi-echo T2 mapping (repetition time: 1510 ms; echo time: 13.8/27.6/41.4/55.2/69 ms ; flip angle: 180°; thickness: 3 mm; field of view: 256 × 264; bandwidth: 225; pixel size: 1 × 1 mm), and (3) multi-echo T2* mapping (repetition time: 428 ms; echo time: 4.35/11.83/19.31/26.79/34.27 ms ; flip angle: 69°; thickness: 3 mm; field of view: 256 × 264; bandwidth: 260; pixel size: 1 × 1 mm). The times of acquisition were 164 s for the T1-weighted fast spin-echo sequence, 231 s for the T2-weighted fast spin-echo sequence, 88 s for the T1 mapping sequence, 306 s for the T2 mapping sequence, and 101 s for the T2* mapping sequence. The nine elements of the spine coil were used to obtain the sequences. An example of the full FOV images for T1, T2, and T2* mapping is provided as Supplementary Data (Figure S1 in the Appendix). To prevent diurnal variations in the relaxation times, as previously described^28^, the images were systematically acquired in the morning.

### Image analysis

The image data were analyzed with Horos software 3.0® (Horos Project, Geneva, Switzerland). Radiographs were useful to determine the number of lumbar IVDs in each sheep, as they vary between 5 and 6 in this species^29^, and to verify the absence of major vertebral disease (e.g., malformations, old fracture, or tumors).

Each IVD was assigned a degenerative score on T2-weighted mid-sagittal images using a 5-point grading system as described by Pfirrmann et al.^9^

**Figure 1:**
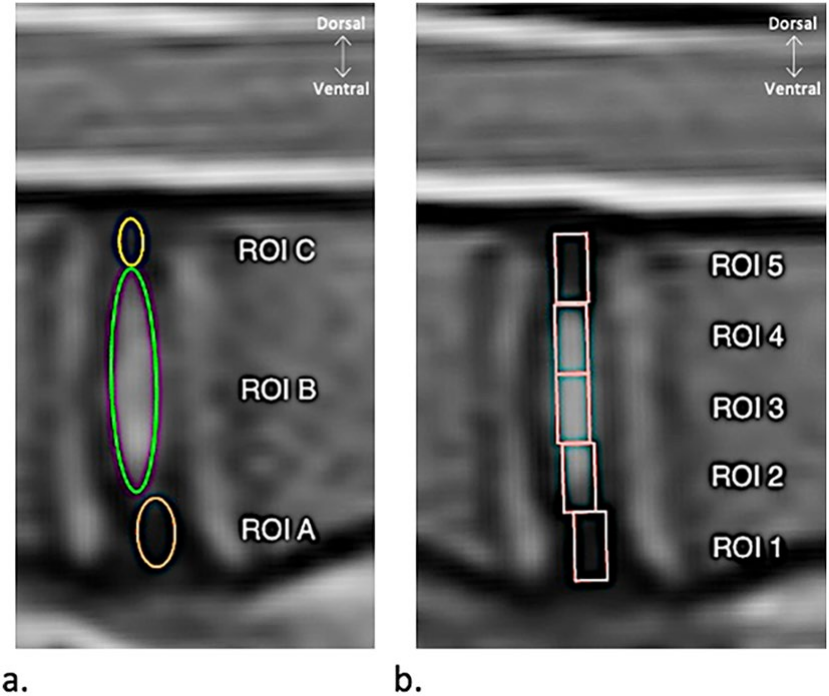
The region-of-interest (ROI) drawing methods used to delimitate the different areas of ovine lumbar discs on mid-sagittal conventional T2-weighted MRI sequences. (**a**) 3-ROIs oval method with ROI A corresponding to the ventral *Annulus fibrosus* (AF), ROI B to the *Nucleus Pulposus* (NP), and ROI C to the dorsal AF. (**b**) 5-ROIs equal-oblong method with ROI 1 corresponding to the ventral AF, ROI 2 to the junction between the ventral AF and the NP, ROI 3 to the NP, ROI 4 to the junction between the NP and the dorsal AF, and ROI 5 to the dorsal AF.

To delineate the various parts of the IVD, ROIs were drawn following two previously described drawing methods: the 3-ROIs method and the 5-ROIs method (Fig. 1)^27^. The size of each ROI varied according to the size of the IVD. In the 3-ROI method, on the T2-weighted images, an oval ROI was manually drawn around the NP (ROI B) and two other oval ROIs were drawn from either side representing the ventral AF (ROI A) and the dorsal AF (ROI C). Particular attention was paid to not include parts of the vertebral endplates in the ROI. The contrast of the images was changed manually in order to distinguish better the NP. In the 5-ROI method, a rectangular ROI was drawn on the T2-weighted images with these limits: between the cranial and the caudal endplates and between the dorsal and the ventral borders of the IVD. This rectangular region was subdivided into 5 equal rectangular ROIs with ROI 1 corresponding to the most ventral ROI and ROI 5 corresponding to the most dorsal ROI. For each IVD, those eight ROIs were copied on the T2 weighted images and pasted on the corresponding T1, T2 and T2* mapping images. The position of each square in the 5-ROI method was manually slightly readjusted in the craniocaudal axis in order not to include vertebral endplates in the ROI section. The mean T1, T2, and T2* relaxation time values of each ROI were collected. Each value was determined by the mapping method (T1, T2, or T2*) and the corresponding letter (3-ROIs method) or number (5-ROIs method). For example, the value of the NP collected by the 3-ROIs method on the T1 mapping images was designated “T1ROIB”. The data of the time dedicated to each drawing method was collected by one observer on one IVD of each animal. The time dedicated was measured from the opening of the qualitative MRI image on which the ROIs were initially drawn to the end of the data collection on the three quantitative mapping sequences. The mean time of the ten measures was kept to compare the two drawing methods.

### Histological analysis

The animals were euthanized after the MR exam by intravenous injection of 140 mg/kg pentobarbital. Fifty lumbar discs were harvested and fixed with 4% paraformaldehyde then placed in a Shandon TBD-2 decalcifier (Thermo Fisher Scientific, USA) and frozen in isopentane chilled with dry ice before being embedded in Super Cryoembedding Medium (Section Lab, Japan). The samples were cut at −30 °C in an axial orientation into 7-μm slices with a CryoStar NX70 (Thermo Fisher Scientific, USA). Following standard protocols, Hematoxylin–Eosin-Safran and Alcian-Blue stainings were performed. Because IVD degeneration is well acknowledged to initiate mainly in the NP^30^, the IVDs were analyzed using modified Boos' scoring focusing only on the NP^31^. Cell density, mucous degeneration, tears and cleft formations, and granular changes were assessed to classify the IDD. The scores ranged from 0 (no degeneration) to 22 (complete degeneration). A blind evaluation of the histological samples was performed separately by two observers. If the scoring differed by less than two points, the median of the two values was used for the analysis. If the scoring differed by more than two points, the histological sample was examined once again by the two raters jointly to reach a consensus.

### Statistical analyses

The statistical analyses were performed with R-software (4.0.3, The R Foundation, Vienna, Austria). Five measurements of the imaging data were collected for each MRI parameter with a 1-week interval between the measurements by two independent blinded observers.

- Intra-rater reliability for the Pfirrmann grading and each ROI on the T1, T2, and T2* mapping sequences: a linear mixed-effects model was used. The intra-rater reliability was considered to be good if the value was < 15% and moderate if the value was between 16 and 50%.
- Inter-rater reliability for the Pfirrmann grading: the weighted kappa coefficient (κ) was used to determine the interobserver reliability for the Pfirrmann grading. The agreement was excellent for κ > 0.80, good for κ = 0.60–0.79, moderate for κ = 0.40–0.59, fair for κ = 0.20–0.39, and slight for κ < 0.2. The five readings were reviewed and the grade that was assigned the most to each IVD by each observer was retained for the determination of the inter-rater reliability.
- Inter-rater reliability for each ROI on the T1, T2, and T2* mapping sequences: when the intra-rater reliability was < 15%, the mean of the five values was retained for the calculation of the inter-rater reliability. A coefficient of determination was used. Reliability was considered to be excellent if R^2^ > 0.75, good if R^2^ = 0.6–0.75, moderate if R^2^ = 0.4–0.59, and poor if R^2^ < 0.4.
- Correlations between the mapping data and the Pfirrmann and the modified Boos' scorings were analyzed using Spearman’s correlation. The correlations were considered significant if the p-value was < 0.05. For each MRI quantitative parameter, significant differences between the Pfirrmann grades were assessed using a linear mixed-effects model followed by comparisons between groups by a Wilcoxon-Mann–Whitney test. The differences were statistically significant when the p-value was < 0.05. For every linear mixed-effects model, the normality and the independence conditions of the residuals were validated according to the method described by Pinheiro and Bates^32^ for mixed-effects models.

## Results

### Intra-observer agreement

The intra-observer analysis revealed strong agreement for all of the parameters (< 15%) except for T2*ROI2. The T2* relaxation time measurement for ROI 2 had moderate intra-observer agreement for observer 1 (20%). Detailed data are provided in Table S1 in the Appendix (Supplementary data).

### Inter-observer agreement

The inter-rater analysis revealed good agreement for the Pfirrmann grading (k = 0.64).

The inter-observer analysis showed strong agreement between the two observers (p < 0.001 and R^2^ > 0.75) for T1, T2, and T2* mapping for all of the NP ROIs (ROIs B and 3) and for the AF/NP junction ROIs (ROIs 2 and 4). The inter-observer agreement was variable for the AF ROIs (ROIs 1, 5, A, and C). It was poor for T2 ROI 1 (p = 0.8, R^2^ = 0.01), T2 ROI 5 (p = 0.55, R^2^ = 0.01), and T2 ROI C (p = 0.66, R^2^ = 0.01), moderate to poor for T1 ROI A (p < 0.05, R^2^ = 0.54) and T2 ROI A (p < 0.05, R^2^ = 0.21), and good for T2* AF ROI (p < 0.05, R^2^ = 0.6–0.73). There was strong agreement between the two observers for the AF only for T1 ROIs 1, 5, and C (p < 0.001 and R^2^ > 0.75). Detailed data are provided in Table S2 in the Appendix (Supplementary data).

As IVD regenerative therapeutic strategies are focused on the NP tissue and as the inter-observer agreement was variable for the AF ROIs, the decision was then made to focus the presentation of the results on the NP ROIs (ROIs 2, 3, 4, and B).

### Imaging analysis

No animal was excluded because of major vertebral disease. Five IVDs were counted in five sheep and six IVDs were counted in the five other sheep.

**Figure 2:**
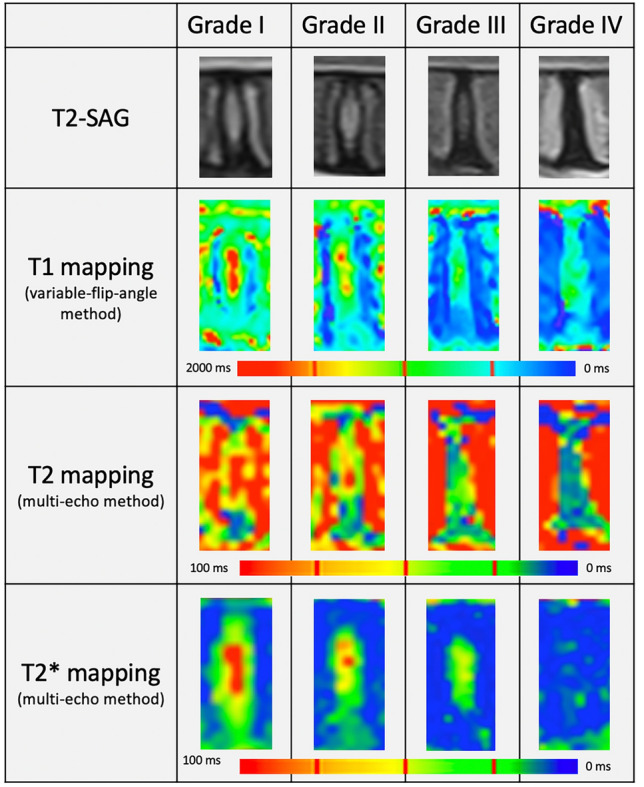
Mid-sagittal T2-weighted sequences and corresponding T1, T2, and T2* maps of lumbar ovine intervertebral discs with different Pfirrmann grades. Each pixel represents the quantitative relaxation time of T1, T2, or T2*. Pfirrmann grade I is identified by a homogeneous section that is isointense to the cerebrospinal fluid representing the *Nucleus pulposus* (NP), with a clear distinction between this structure and the *Annulus fibrosus* (AF). On the map images, the NP has a high relaxation time value, represented in red images on the color maps, while the AF, which is less hydrated, has low green relaxation time values. As the degenerative process involves a loss of the macromolecular structure of the disc and a decrease in its water content, the relaxation times in the NP and the AF decline. Pfirrmann grade IV is identified by an inhomogeneous central section, hypointense to the cerebrospinal fluid, with a loss of distinction between the NP and the AF and low relaxation time values in the AF and the degenerated NP, for which the pixels appear blue or green on the color maps.

Regarding the Pfirrmann grading, 11 IVDs (22%) were classified as grade I, 17 (34%) as grade II, 16 (32%) as grade III, and 6 (12%) as grade IV. None were classified as grade V. The mean relaxation time values of the ten measurements for the different NP ROIs for each mapping method and for each Pfirrmann grade are summarized in Table 1 and illustrated in Fig. 2.

**Table 1:** The mean quantitative T1 (a), T2 (b), and T2* (c) relaxation times and the standard deviations (SD) of the *Nucleus pulposus* (NP) regions-of-interests (ROIs) of lumbar ovine intervertebral disc for each Pfirrmann grade (in milliseconds).

	Grade I (n = 11)		Grade II (n = 17)		Grade III (n = 16)		Grade IV (n = 6)	
	Mean	SD	Mean	SD	Mean	SD	Mean	SD
(a)								
T1 ROI B	1268.3	349.1	1308	156.7	701.5	291.6	676.7	198.9
T1 ROI 2	998.4	222.3	1001	129.5	541.7	196.8	586.4	162.2
T1 ROI 3	1381.9	299.9	1514.8	258.8	785.7	316.6	712.3	221.1
T1 ROI 4	1285.8	267.1	1357.5	232.8	742.9	344.7	667.3	182.4
(b)								
T2 ROI B	89.8	18.8	132.9	39.7	86	27.6	29.3	22.9
T2 ROI 2	60	29.7	91	67.7	56.1	24.7	23.3	19.8
T2 ROI 3	104.6	24.5	185.4	71.1	107	54.3	29.1	29.2
T2 ROI 4	96.9	36.7	167.4	69.3	79.7	41.8	27.5	13.9
(c)								
T2* ROI B	42.3	4.5	54.2	11.6	38.6	9.7	12.5	6.5
T2* ROI 2	33.4	9.5	43.9	12.9	29.5	10.2	12.2	5.4
T2* ROI 3	53.8	10.8	71.9	18.5	49.2	18.4	12.7	9.4
T2* ROI 4	46.9	14.1	64.3	23	36.8	14.2	11.7	6.5

The Spearman correlations showed that the Pfirrmann grading was negatively correlated with the NP values measured with ROIs 2, 3, 4, and B on the T1 mapping images (Table 1a), the T2 mapping images (Table 1b), and the T2* mapping images (Table 1c) (p < 0.05).

In terms of the T1 relaxation time measurements, statistically significant differences were found between Pfirrmann grades II and III (p < 0.001), I and III (p < 0.05), I and IV (p < 0.05), and between II and IV (p < 0.05) for all of the NP ROIs. By contrast, there was not a significant difference between grades I and II and between grades III and IV for any of the four NP T1 ROIs (Table 2a).

**Table 2:** The results of the post-hoc Wilcoxon-Mann–Whitney test (*p-values*) comparing the different Pfirrmann grades for each *Nucleus pulposus* (NP) region-of-interest (ROI) assessed with (a) T1, (b) T2, and (c) T2* mapping. P-values < 0.05 are highlighted in bold.

			I/II	II/III	III/ IV	I/III	I/IV	II/IV
(a)	T1 values	ROI B	0.8106	** < 0.0001**	0.7778	**0.0002**	**0.0018**	** < 0.0001**
		ROI 2	0.6788	** < 0.0001**	0.4613	** < 0.0001**	**0.0005**	** < 0.0001**
		ROI 3	0.3938	** < 0.0001**	0.8214	** < 0.0001**	**0.0005**	** < 0.0001**
		ROI 4	0.5268	** < 0.0001**	0.6925	**0.0005**	**0.0002**	** < 0.0001**
(b)	T2 values	ROI B	**0.0016**	** < 0.0001**	**0.0001**	0.7043	**0.0003**	** < 0.0001**
		ROI 2	**0.0339**	**0.0039**	**0.0013**	0.8889	**0.0026**	**0.0002**
		ROI 3	** < 0.0001**	**0.0003**	**0.0007**	0.4837	**0.0003**	** < 0.0001**
		ROI 4	**0.0006**	** < 0.0001**	**0.0001**	**0.0317**	** < 0.0001**	** < 0.0001**
(c)	T2* values	ROI B	**0.0092**	**0.0003**	** < 0.0001**	0.2355	** < 0.0001**	** < 0.0001**
		ROI 2	**0.038**	**0.0001**	** < 0.0001**	0.2693	** < 0.0001**	** < 0.0001**
		ROI 3	**0.0069**	**0.0007**	** < 0.0001**	0.1642	** < 0.0001**	** < 0.0001**
		ROI 4	0.0655	** < 0.0001**	** < 0.0001**	**0.0435**	** < 0.0001**	** < 0.0001**

Analysis of the T2 relaxation time measurements revealed statistically significant differences between all of the Pfirrmann grades except between grades I and III for T2 ROIs 2, 3, and B. Only T2 ROI 4 had significant differences between all of the Pfirrmann grades (Table 2b).

Similarly, for the T2* relaxation time measurements, statistically significant differences were found between all of the Pfirrmann grades except between grades I and III for T2* ROIs 2, 3, and B. For T2 ROI 4, there were statistically significant differences between all of the Pfirrmann grades except between grades I and II (Table 2c).

### Correlation between the MRI relaxation time measurements and the modified Boos’ scoring

The histological scores ranged from three for the healthiest IVD to nineteen for the most degenerated one. The mean value was 9.4 and the median value was 9. Graphical distribution of the histological scores of the IVDs is provided in Figure S2 in the Appendix (Supplementary data).

**Figure 3:**
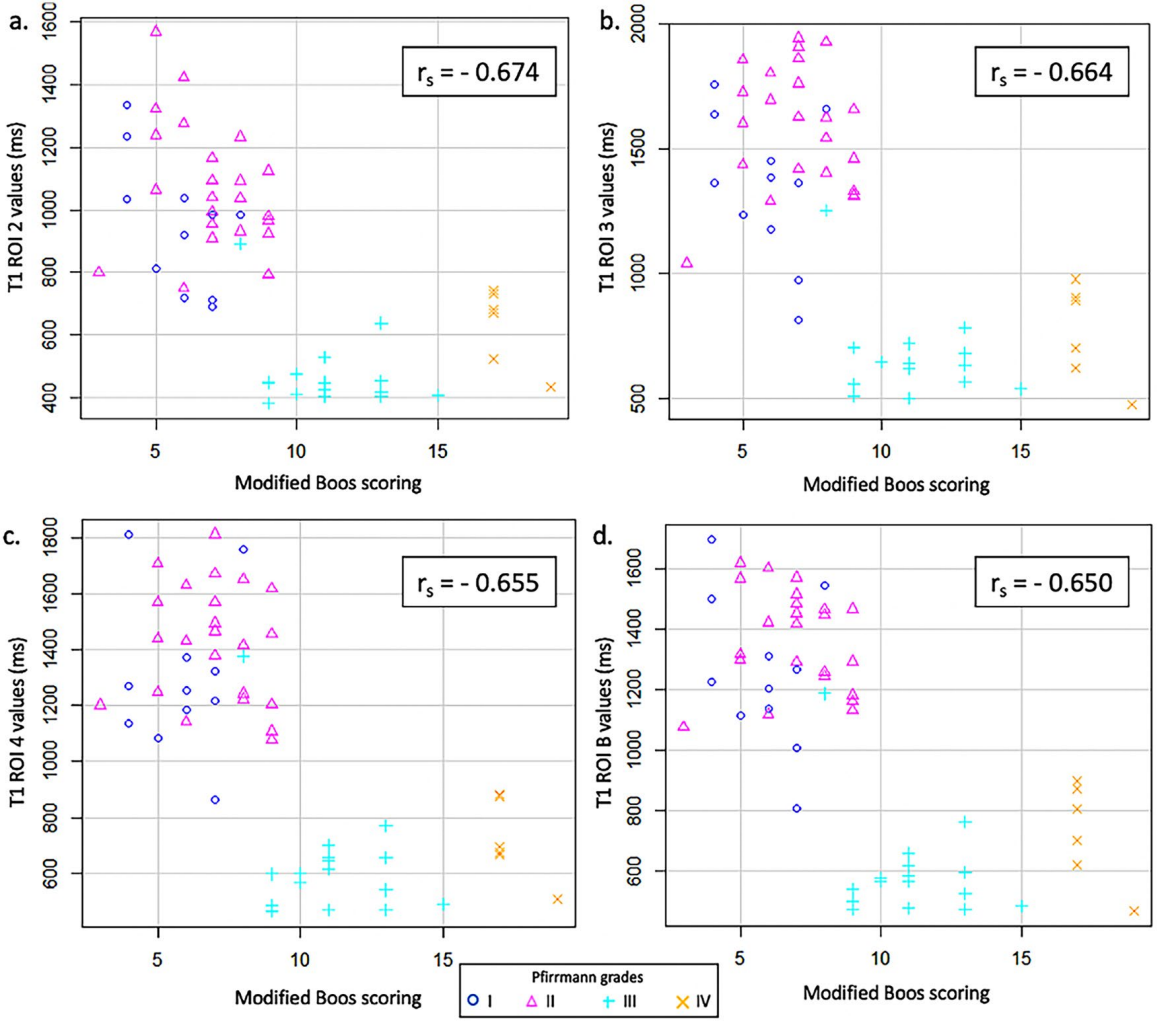
T1 relaxation time values in the *Nucleus pulposus* (NP) of ovine intervertebral discs according to the modified Boos’ score and the Pfirrmann grade. The mean T1 relaxation time of the NP was measured by regions-of-interest (ROIs) that included the NP or a part of the NP of each lumbar ovine disc on the sagittal T2-weighted image and by copying this ROI onto the corresponding T1 mapping image. Equal-oblong ROIs delimitated (**a**) the junction between the NP and the ventral *Annulus fibrosus* (AF) (T1 ROI 2), (**b**) the NP (T1 ROI 3), and (**c**) the junction between the NP and the dorsal AF (T1 ROI 4). (**d**) A manually drawn oval delineated the NP (T1 ROI B). The modified Boos’ scoring was negatively correlated with the NP values measured for ROIs 2, 3, 4, and B on the T1 mapping images (p < 0.001).

**Figure 4:**
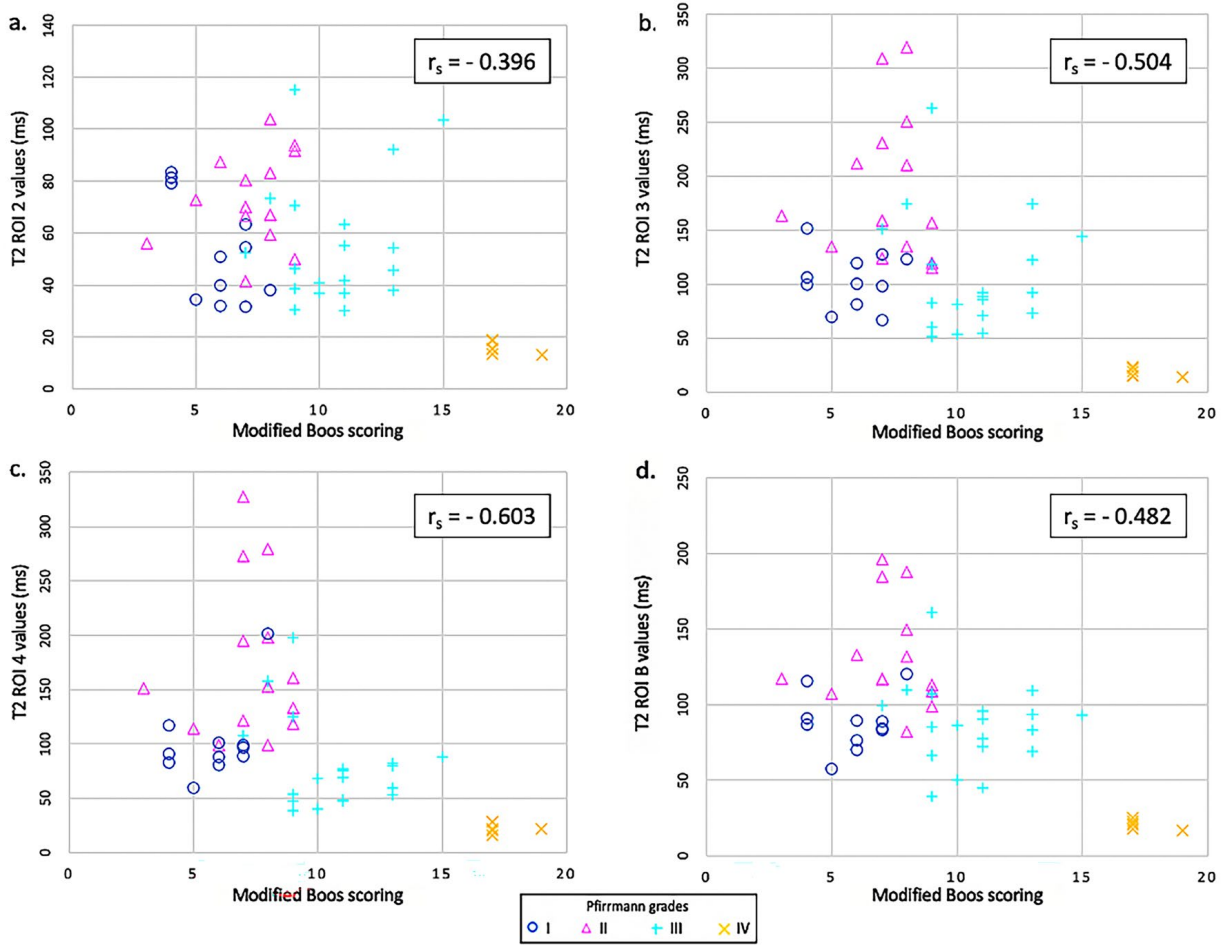
T2 relaxation time values in the *Nucleus pulposus* (NP) of ovine intervertebral discs according to the modified Boos’ score and the Pfirrmann grade. The mean T2 relaxation time of the NP was measured by regions-of-interest (ROIs) that included the NP or a part of the NP of each lumbar ovine disc on the sagittal T2-weighted image and by copying this ROI onto the corresponding T2 mapping image. Equal-oblong ROIs delimitated (**a**) the junction between the NP and the ventral *Annulus fibrosus* (AF) (T2 ROI 2), (**b**) the NP (T2 ROI 3), and (**c**) the junction between the NP and the dorsal AF (T2 ROI 4). (**d**) A manually drawn oval delineated the NP (T2 ROI B). The modified Boos’ scoring was negatively correlated with the NP values measured for ROIs 2, 3, 4, and B on the T2 mapping images (p < 0.001).

**Figure 5:**
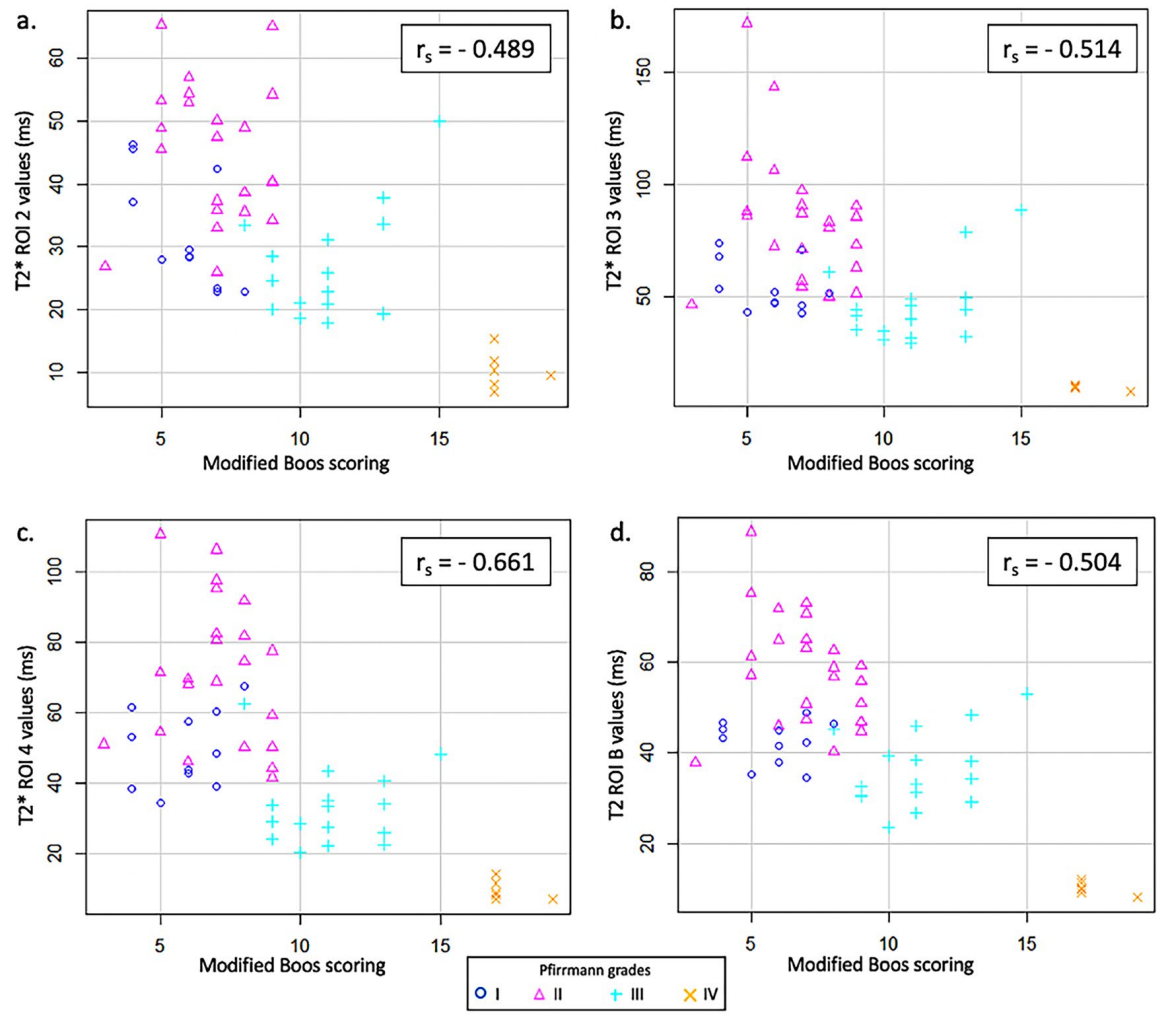
T2* relaxation time values in the *Nucleus pulposus* (NP) of ovine intervertebral discs according to the modified Boos’ score and the Pfirrmann grade. The mean T2* relaxation time of the NP was measured by regions-of-interest (ROIs) that included the NP or a part of the NP of each lumbar ovine disc on the sagittal T2-weighted image and by copying this ROI onto the corresponding T2* mapping image. Equal-oblong ROIs delimitated (**a**) the junction between the NP and the ventral *Annulus fibrosus* (AF) (T2* ROI 2), (**b**) the NP (T2* ROI 3), and (**c**) the junction between the NP and the dorsal AF (T2* ROI 4). (**d**) A manually drawn oval delineated the NP (T2* ROI B). The modified Boos’ scoring was negatively correlated with the NP values measured for ROIs 2, 3, 4, and B on the T2* mapping images (p < 0.001).

The Spearman correlations showed that the modified Boos’ scoring was negatively correlated with the NP values measured for ROIs 2, 3, 4, and B on the T1 mapping images (Fig. 3), the T2 mapping images (Fig. 4), and the T2* mapping images (Fig. 5) (p < 0.05). Histological illustrations of healthy, slightly, and severely degenerated NP and their corresponding imaging data are provided in Figures S3 and S4 in the Appendix (Supplementary data).

### Comparison between the two methods of drawing ROIs for clinical application

Considering the ROI B from the 3-ROIs method and the three NP ROIs from the 5-ROIs method together, we found that the two methods were quite similar for evaluation of the NP relaxation time values on T1, T2, and T2* mapping images.

Considering the time required for each method, the mean time for the 3-ROIs method was much faster than the 5-ROIs method (50 s vs. 120 s). These times do not take into account the selection of the two sequences and their correct display on the screen, which do not vary from one method to the other. The number of computer manipulations was much higher for the 5-ROIs method compared to the 3-ROIs approach (22 vs. 11).

### Pfirrmann score probabilities

**Figure 6:**
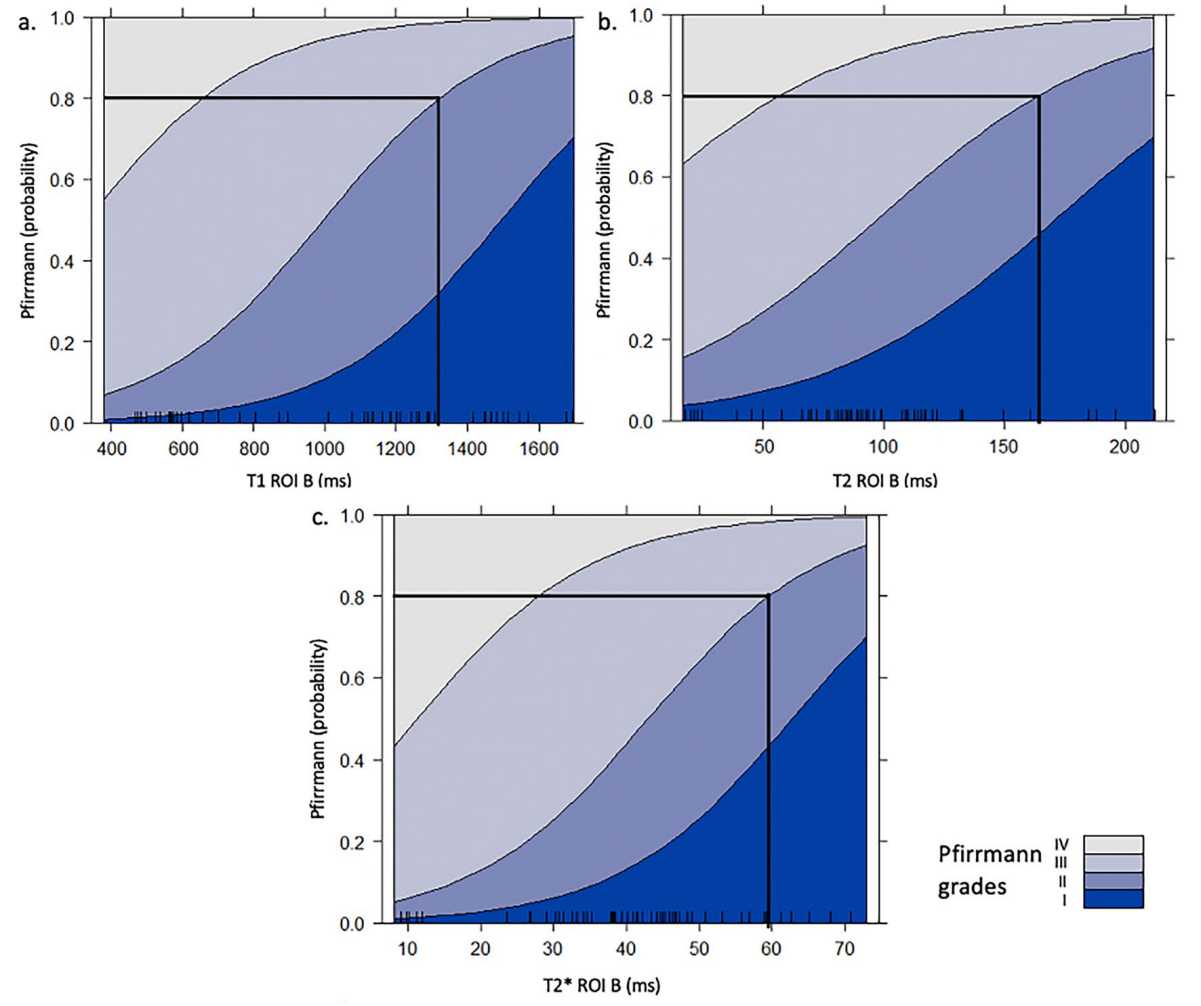
Pfirrmann score probabilities for the manually drawn region-of-interest (ROI) that delimitates the *Nucleus pulposus* (NP) designated ROI B on the MR quantitative (**a**) T1, (**b**) T2, and (**c**) T2* mapping sequences. (**a**) If the T1 ROI B relaxation time value exceeds 1,320 ms, the probability of the NP being scored a Pfirrmann grade I or II is over 0.8. (**b**) If the T2 ROI B relaxation time value exceeds 164 ms, the probability of the NP being scored a Pfirrmann grade I or II is over 0.8. (**c**) If the T2* ROI B relaxation time value exceeds 59 ms, the probability of the NP being scored a Pfirrmann grade I or II is over 0.8.

With an ordinal regression, the probability of the degenerative degree according to the relaxation time value was obtained for T1 ROI B, T2 ROI B, and T2* ROI B and are illustrated in Fig. 6.

Considering ROI B, the probability of a non-degenerated NP (Pfirrmann grade I) or an NP with few degenerative changes (Pfirrmann grade II) is higher than 80% if the T1, T2, and T2* relaxation times are greater than 1,320 ms, 164 ms, and 59 ms, respectively.

## Discussion

This study investigated the effectiveness and the feasibility of MR T1, T2 and T2* mapping MRI using two drawing ROI methods on the ovine lumbar IVD. These sequences were correlated to qualitative MR Pfirrmann grade and histological scoring in order to find the most suitable approach that can readily be used in practice in veterinary and human medicine. In accordance with previous studies, most of the AF ROIs offer poor-to-moderate inter-agreement, contrary to the NP ROIs, irrespective of the MRI quantitative sequences^27,33^. The manually drawn oval ROI A and ROI C representing the ventral and the dorsal AF, respectively, were assessed subjectively. The position of the rectangular ROI 1 and ROI 5 representing the ventral and the dorsal AF, respectively, in the 5-ROI method were also adjusted manually. Although particular care was taken to draw an oval ROI of maximal size or to replace the rectangular ROIs without including the border of the NP and the vertebral endplates, a degree of variation may remain, and this could explain the lack of agreement between the two observers for this ROI. This result does not influence the assessment and the quantification of early degeneration of the NP, when MR qualitative grading systems fail to reach this goal with adequate precision^7^. Indeed, as the early events of IDD happen in the NP, it is crucial to find a non-invasive technique to detect the subtle changes that occur in this tissue. Normative values for healthy adult ovine lumbar IVD have only been established for the T2* mapping on 5 IVDs. Mean T2* relaxation time values for the ventral NP, the central NP, and the dorsal NP were 31.8, 44.1, and 37.3 ms, respectively^19^, which is coherent with the T2* values obtained in this study. Numerous studies have focused on the correlation between T2 and T2* times with various parameters to characterize IDD. A strong negative correlation has been found between T2 and T2* times and MR qualitative Pfirrmann grading of IDD, which is consistent with our results^10,12,20,33–37^. In contrast, T1 mapping has rarely been used to assess IDD, although it has been studied extensively for other tissues’ perfusion such as brain or myocardium^38^. Very few studies have applied T1 mapping sequences to the IVD, all of which used the multiple-inversion-recovery method^28,36,39,40^. Our study, to the best of our knowledge, is the first to utilize the variable-flip-angle method and to demonstrate the strong correlation between qualitative MR and histology. The main difference between the two acquisition methods is the time of acquisition of the sequences. The number of inversion-recuperation sequences varies between 5 and 16. Only one of those studies provides the actual time of acquisition of each sequence^28^, in which the total time of acquisition of the T1 mapping was 784 s, while our method only took 88 s, thus allowing for time savings in daily practice.

Relaxation time measurement is a real challenge in terms of the risk of bias. In this study, great care was taken to reproduce the MR acquisitions under similar conditions. Diurnal significant variation has been highlighted previously in the NP using T2^41,42^ and T1 mapping^28^ in healthy human lumbar IVD due to the decrease in water content that occurs during a diurnal cycle. One study has assessed this variation in the human IVD using T2* mapping but no significant differences have been identified (p-value = 0.748)^43^. In sheep, a potential diurnal variation in mapping values has not been investigated yet but, for ethical considerations, we decided not to anesthetize our cohort twice in one day to explore this feature and realized all the MR exams in the morning. In in vivo studies, quantitative MRI is also sensible to motion artifacts induced by respiration and involuntary displacements of the specimen during the acquisition of the sequences^39^ but the fact that our study was conducted on anesthetized sheep put under mechanical ventilation limited the presence of these artifacts. Moreover, mapping sequences may be prone to artifacts. When selecting the FOV of each mapping sequence, particular care was taken to select not only the lumbar IVDs but to extend to the last thoracic IVDs and the lumbosacral junction. This allowed spatial warping at the border of the images to be avoided and thus limit such artifacts.

As there is no standardized method for ROI acquisition, comparison of mapping data is not straightforward. Numerous approaches are used to assess IVD degeneration that focus on various parts of the IVD. In accordance with the study that compared three ROI drawing methods^27^, our data confirm the moderate-to-poor inter-observer agreement for AF ROIs, unlike NP ROIs. No pertinent difference was found between the two approaches used in our study to define the NP and its junction with the AF. Although ROI 4 appears to be more precise at distinguishing the degenerative grade, especially on T2 images, the drawing method to obtain this ROI implies drawing the 5 ROIs together before isolating the mean value obtained with ROI 4. On the other hand, in the 3-ROIs method, the ROIs are independent and can be drawn individually. In clinical practice, the mean time required to obtain ROI 4 is then greater than for ROI B (150 vs. < 50 s). Moreover, the 5-ROIs method appears to be more complicated than the other method in terms of software manipulations and thus involves a steeper learning curve, especially for an untrained observer. Therefore, of the three mapping methods, ROI B appears to be the most suitable tool for evaluation of the NP, and it warrants being assessed for routine clinical practice in human medicine. ROI B has many benefits: ease of drawing, timeliness, and sensitivity.

Even if a perfect match with human IVD degeneration can’t be found, animal models are crucial in studies related to degeneration or regeneration therapeutic strategies of this tissue, and, notably, spontaneous degenerative models^23,24,26^. In particular, ovine lumbar spine and discs present a lot of similarities with human tissues regarding the anatomy, biology, and biomechanics^44–46^. Preclinical investigations with intradiscal injections are essential to test the new therapeutics developed in vitro before clinical transposition to human patients. Surprisingly, if sheep have been well described in IVDD preclinical studies, very few investigations using quantitative MRI have been conducted on this species. T2* mapping and T2 mapping have been negatively correlated with disc degeneration using modified Boos^19^ and non-enzymatic glycation^47^, respectively. T2 reference values according to the sheep age have been established in a study that used MRI and CT to assess the spontaneous disc degeneration^25^. Our data indicated that T1, T2, and T2* mapping are applicable in sheep to estimate the changes occurring in the NP in preclinical regenerative studies focusing on this tissue.

Finally, few studies have assessed the correlation between in vivo quantitative MR measurements and histology of the NP. Investigations using animal models facilitate the comparison of imaging modalities and histology. Boos’ scoring has been used in a T2* mapping study of the lumbar ovine NP of healthy IVDs, but not for correlation^19^. Our study is the first, to our knowledge, to demonstrate the correlation between T1, T2, and T2* mapping and histology in an ovine model of lumbar IVD degeneration. Imaging may be a valuable substitute for histological analysis of the ovine IVD, by limiting the number of animals sacrificed in accordance with the 3R-ethics-rules. Moreover, longitudinal assessment of the IVD is likewise useful to study the changes that occur in early IDD. However, while T2 and T2* mapping have been correlated to the water content and molecular interactions^11,14^ and to the spatial architecture of the macromolecules, respectively, further studies are needed to establish which molecular changes the T1 value reflects in the IDD process.

In conclusion, quantitative T2, T2*, and T1 mapping using a single manual oval ROI focused on the NP are easy, reliable, time-efficient, and reproducible tools to evaluate lumbar IVD degenerative disease in sheep and they could be useful tools for routine clinical practice. Early detection and characterization of IVD degeneration will need such tools to assist with the diagnosis and to help with the therapeutic decision. In particular, variable-flip-angle T1 mapping appears to be a relevant modality for assessment of the pathological changes in the lumbar IVDs and it warrants particular consideration in further preclinical and clinical studies.

## Supplementary Information

Supplementary Information.

## Data Availability

The data and materials underlying this article will be shared on request to any of the corresponding authors.

## Abbreviations

LBP: Low Back Pain
IVD: Intervertebral Disc
IDD: Intervertebral Disc Degeneration
NP: *Nucleus pulposus*
AF: *Annulus fibrosus*
ROI: Region-of-interest
