# Supplementary material for: Comparison of MRI T1, T2, and T2* mapping with histology for assessment of intervertebral disc degeneration in an ovine model
Source: Sci Rep. 2022 Mar 30;12:5398. doi: 10.1038/s41598-022-09348-w (PMC8967912; doi:10.1038/s41598-022-09348-w)

Comparison of MRI T1, T2, and T2* mapping with histology for assessment of intervertebral disc degeneration in an ovine model.

Supplementary Information

Nora Bouhsina[1,2,3], Cyrille Decante[1,2,4], Jean-Baptiste Hardel[3], Dominique Rouleau[1,3], Jérôme Abadie[5,6], Antoine Hamel[1,2,4], Catherine Le Visage[1,2], Julie Lesoeur[1,2], Jérôme Guicheux*[1,2,7], Johann Clouet[$1,8,9], and Marion Fusellier[$1,2,3]

[1] INSERM, UMRS 1229, Regenerative Medicine and Skeleton (RMeS), Université de Nantes, ONIRIS, Nantes, F-44042, France

[2] Université de Nantes, UFR Odontologie, Nantes, F-44042, France

[3] Department of Diagnostic Imaging, CRIP, ONIRIS, College of Veterinary Medicine, Food Science and Engineering, Nantes, F-44307, France

[4] CHU Nantes, Service de Chirurgie Infantile, PHU5, Nantes, F-44093, France

[5] Laboniris, ONIRIS, College of Veterinary Medicine, Food Science and Engineering, Nantes, F-44307, France

[6] CRCINA, INSERM, Université d'Angers, Université de Nantes, Nantes, France

[7] CHU Nantes, PHU4 OTONN, Nantes, F-44093, France

[8] Université de Nantes, UFR des Sciences Biologiques et Pharmaceutiques, Nantes, F-44042, France

[9] CHU Nantes, Pharmacie centrale, PHU11, Nantes, F-44042, France

* Corresponding author: jerome.guicheux@inserm.fr, +33 240412916

$ Co-last authors

a.	Observer 1	Observer 2
Pfirrmann grading	**13**	**9**

b.	Observer 1	Observer 2
T1 ROI A	**< 1**	**< 1**
T1 ROI B	**< 1**	**< 1**
T1 ROI C	**< 1**	**< 1**
T1 ROI 1	**< 1**	**< 1**
T1 ROI 2	**< 1**	**< 1**
T1 ROI 3	**< 1**	**< 1**
T1 ROI 4	**< 1**	**< 1**
T1 ROI 5	**< 1**	**< 1**

c.	Observer 1	Observer 2
T2 ROI A	**8**	**2**
T2 ROI B	**5**	**1**
T2 ROI C	**8**	**10**
T2 ROI 1	**3**	**5**
T2 ROI 2	**< 1**	**< 1**
T2 ROI 3	**< 1**	**1**
T2 ROI 4	**2**	**4**
T2 ROI 5	**9**	**11**

d.	Observer 1	Observer 2
T2* ROI A	**15**	**2**
T2* ROI B	**3**	**4**
T2* ROI C	**2**	**14**
T2* ROI 1	**< 1**	**1**
T2* ROI 2	20^{+}	**6**
T2* ROI 3	**11**	**3**
T2* ROI 4	**3**	**7**
T2* ROI 5	**5**	**10**

Table S1: Linear mixed-effects model for intra-agreement analysis for the Pfirrmann grading (a.), T1 mapping (b.), T2 mapping (c.), and T2* mapping (d.) using the 3-ROIs and the 5-ROIs drawing methods.

Intra-rater reliability is considered to be good if the value is less than 15% (*in bold*) and moderate if the value is between 16 and 50 % ($^{+}$).

Good intra-rater agreement was found for all parameters except for the anterior and posterior IVD height on T1- and T2-weighted images (> 89% for both observers). The T2* relaxation time measurement for the ROI 2 revealed moderate intra-observer agreement for observer 1 (20%).

	p-value	R^2
T1 ROI A	< 0.001	0.55
T1 ROI B	< 0.001	**0.80**
T1 ROI C	< 0.001	**0.81**
T1 ROI 1	< 0.001	**0.87**
T1 ROI 2	< 0.001	**0.85**
T1 ROI 3	< 0.001	**0.91**
T1 ROI 4	< 0.001	**0.91**
T1 ROI 5	< 0.001	**0.82**

	p-value	R^2
T2 ROI A	< 0.001	0.21
T2 ROI B	< 0.001	**0.91**
T2 ROI C	*0.66*	0.01
T2 ROI 1	*0.81*	0.01
T2 ROI 2	< 0.001	**0.75**
T2 ROI 3	< 0.001	**0.93**
T2 ROI 4	< 0.001	**0.84**
T2 ROI 5	*0.55*	0.01

	p-value	R^2
T2* ROI A	< 0.001	0.72 $^+$
T2* ROI B	< 0.001	**0.88**
T2* ROI C	< 0.001	0.62 $^+$
T2* ROI 1	< 0.001	0.64 $^+$
T2* ROI 2	< 0.001	**0.78**
T2* ROI 3	< 0.001	**0.94**
T2* ROI 4	< 0.001	**0.80**
T2* ROI 5	< 0.001	0.73$^+$

Table S2: Coefficient of determination for inter-agreement analysis for T1 mapping (A), T2 mapping (B), and T2* mapping (C) using the 3-ROI and the 5-ROIs drawing methods.

Inter-rater reliability is considered to be excellent if R^2 is above 0.75 (in bold), good if R^2 is between 0.6 and 0.75 ($^+$), moderate if R^2 is between 0.4 and 0.59, and poor if R^2 is under 0.4.

<u>Figure S1</u>:

Full FOV images of the sagittal (a.) T1, (b.) T2, and (c.) T2* mapping sequences in a 2-year-old sheep. When selecting the FOV of each mapping sequence, particular care was taken to select not only the lumbar IVDs but to extend to the last thoracic IVDs and the lumbosacral junction in order to avoid spatial warping at the border of the images.

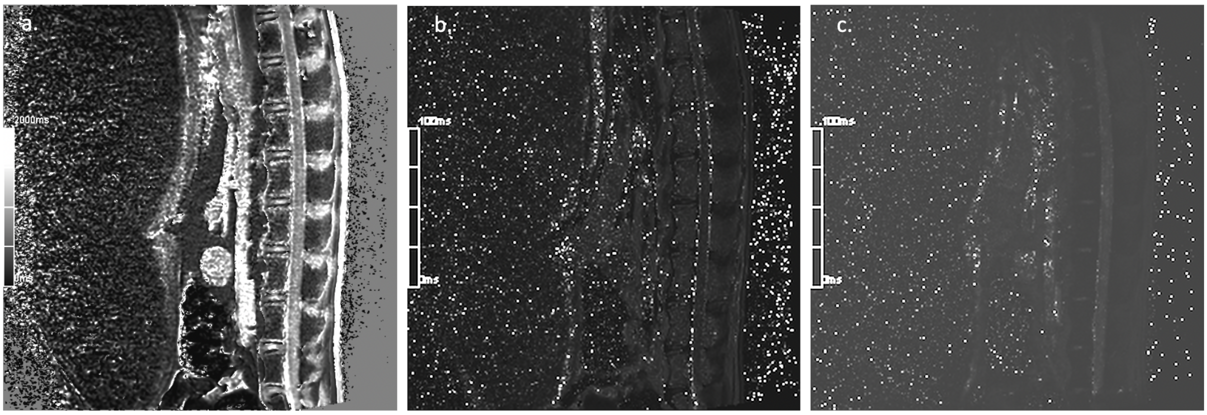

<u>Figure S2</u>:

Graphical distribution of the histological modified Boos' scores of the fifty ovine intervertebral discs.

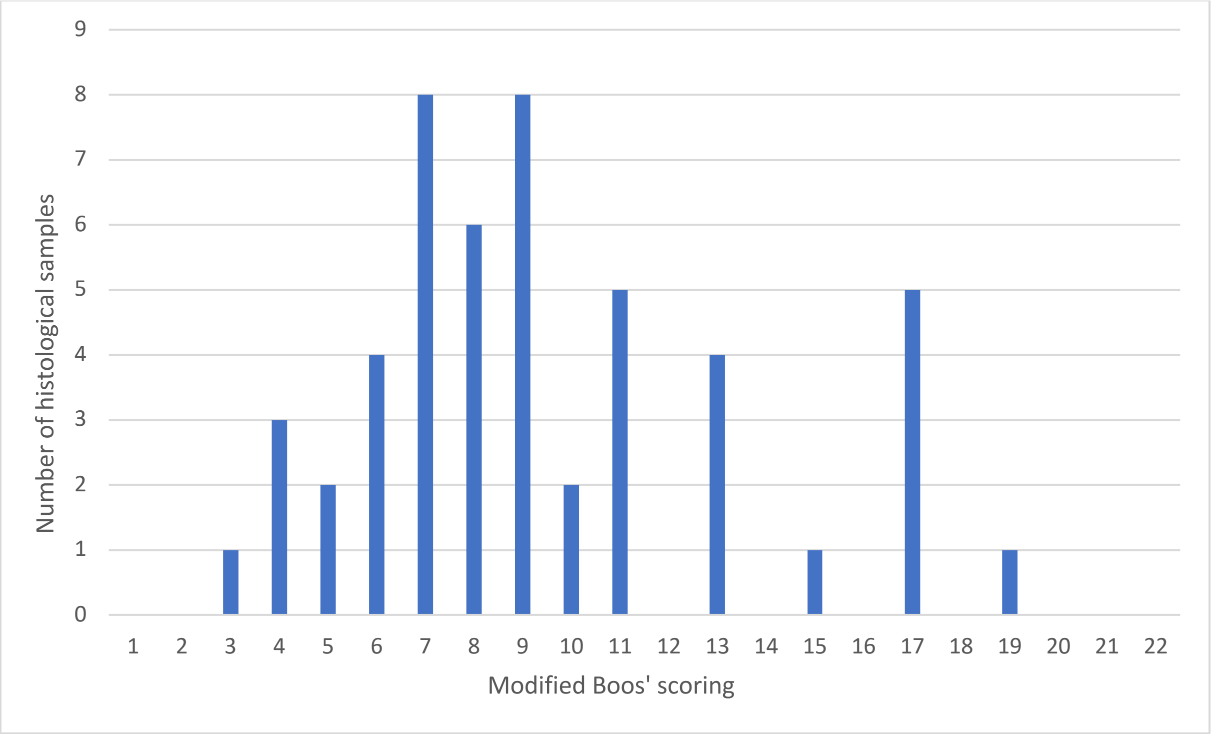

Figure S3:

Histological assessment of ovine lumbar *Nucleus Pulposus* (NP) degeneration and correlation with imaging data.

Histological analysis by Hematoxylin Eosin Safran (a., c.) and Alcian blue (b., d.) staining of lumbar ovine NP according to different grades of degeneration: (a., b.) slightly degenerated NP with a modified Boos score of 3 and (c., d.) severely degenerated NP with a modified Boos score of 17. In the slightly degenerated NP, few cell proliferation (arrow head) and ghost cell (arrow) are present on the Hematoxylin Eosin Safran staining. In the severely degenerated NP, almost no cells are observed. Mucous degeneration appeared as pink areas with Alcian Blue staining and are abundantly present in the severely degenerated NP (stars). The histological healthier NP corresponded to a Pfirrmann score of 3 on T2-weighted images whereas the more severely degenerated NP was given a higher Pfirrmann score of 17. In the same way, the T1, T2 and T2* relaxation time values of the NP delineated manually (ROI B) were much higher for the slightly degenerated NP contrary to the severely degenerated NP.

<table>
<tr><td></td><td colspan="3">Slightly degenerated lumbar IVD</td><td colspan="3">Severe degenerated lumbar IVD</td></tr>
<tr><td>Hematoxylin Eosin Safran (HES)</td><td colspan="3">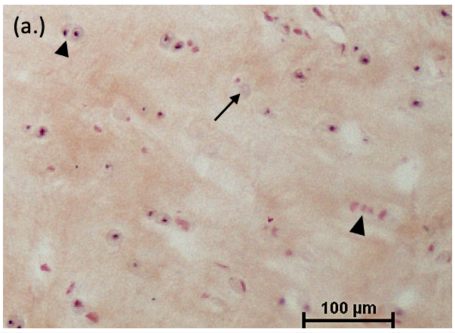
</td><td colspan="3">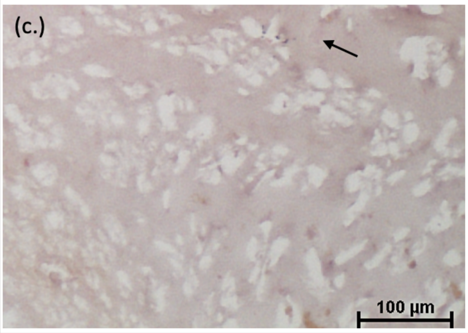
</td></tr>
<tr><td>Alcian Blue (AB)</td><td colspan="3">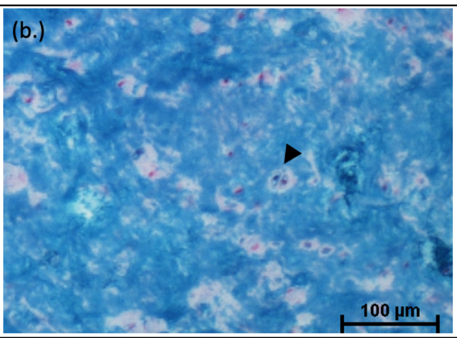
</td><td colspan="3">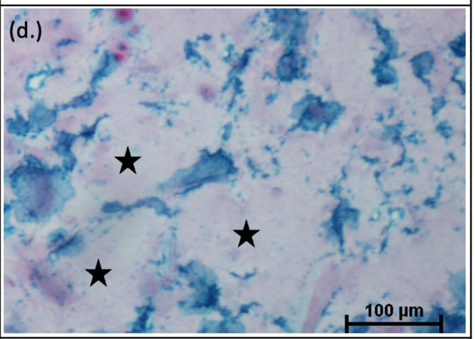
</td></tr>
<tr><td>Pfirrmann grade</td><td colspan="3">**2**</td><td colspan="3">**4**</td></tr>
<tr><td rowspan="2">Relaxation time values (ms)</td><td>T1 ROI B</td><td>T2 ROI B</td><td>T2* ROI B</td><td>T1 ROI B</td><td>T2 ROI B</td><td>T2* ROI B</td></tr>
<tr><td>**1075**</td><td>**117**</td><td>**38**</td><td>**803**</td><td>**20**</td><td>**12**</td></tr>
</table>

Figure S4:

Comparison of sagittal T1, T2, and T2* mapping and axial histological slices of ovine lumbar intervertebral disc (IVD) graded Pfirrmann I and Pfirrmann IV.

With degeneration, the water and the proteoglycan contents of the NP decrease while the content in type I collagen increases. As a consequence, the T1, T2, and T2* relaxation time measurements decrease while the Pfirrmann grades increase. Histologically, with Alcian Blue staining, it leads to mucous degeneration (pink areas in the NP) and the loss of a clear margin between the NP and the AF.

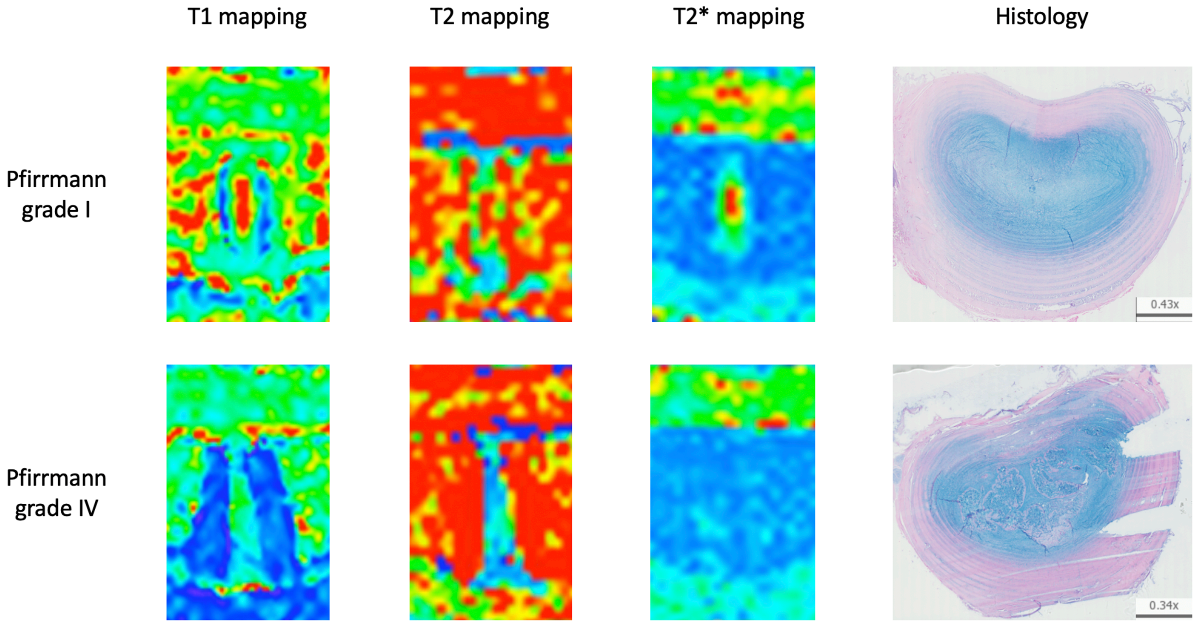

Supplement: Supplementary file 1 — Supplementary Information. [file 41598_2022_9348_MOESM1_ESM.pdf]
